# Transforming maternal and newborn health social norms and practices to increase utilization of health services in rural Bangladesh: a qualitative review

**DOI:** 10.1186/s12884-015-0501-8

**Published:** 2015-03-29

**Authors:** Fahmida Taleb, Janet Perkins, Nabeel Ashraf Ali, Cecilia Capello, Muzahid Ali, Carlo Santarelli, Dewan Md Emdadul Hoque

**Affiliations:** Center for Child and Adolescent Health, icddr,b, 68, Shaheed Tajuddin Ahmed Sarani, Mohakhali, Dhaka 1212 Bangladesh; Enfants Du Monde, Geneva, Switzerland; Enfants Du Monde South Asia Field Office, Dhaka, Bangladesh

**Keywords:** Maternal health, Newborn health, Birth preparation, Community based interventions, Traditional birth attendants, Bangladesh

## Abstract

**Background:**

Since 2008, Participatory Action for Rural Development Innovation (PARI) Development Trust, with the support of Enfants du Monde, has been implementing a maternal and newborn health (MNH) program based on the World Health Organization’s (WHO) framework for Working with Individuals, Families and Communities (IFC) to improve MNH in Netrokona district, Bangladesh. This program aims to empower women and families and increase utilization of quality health services, thereby helping women realize their rights related to maternal health. Birth preparedness and complication readiness and working with traditional birth attendants (TBAs) to exercise a new role in MNH and have formed key interventions of this program. The purpose of this study was to explore how the program has contributed to changing social norms and practices surrounding MNH at midpoint.

**Methods:**

This study relied primarily on qualitative data collection. Two focus group discussions (FGDs) were conducted with women who were pregnant or had recently given birth and one FGD with each of the following groups: husbands, family members, TBAs, and health workers. In-depth interviews were conducted with women who were pregnant or had recently given birth, family members of these women, health care providers, TBAs and community health workers in selected intervention areas.

**Results:**

Since implementation of interventions informants report an increase in planning for birth and complications and a shift in preference toward skilled care at birth. However, women still prefer to receive services at home. TBAs report encouraging women to access skilled care for both routine and emergency services. While community members’ understanding of rights related to maternal health remains limited, they report increased women’s participation in household decision- making processes, an important indicator of the realization of rights.

**Conclusion:**

Results suggest that community-level interventions aiming to affect change in social norms and practices surrounding MNH can influence knowledge and practices even after a short period of time. Further evaluations will be required to quantify the degree to which these changes are having an impact on health services utilization.

## Background

### Maternal and newborn health in Bangladesh

Bangladesh is among the many countries that have struggled to reduce the risks faced by mothers and newborns. Despite significant progress, the most recent maternal mortality ratio (MMR) estimate of 194 maternal deaths per 100,000 live births remains significantly higher than the developed world [1]. Moreover, while impressive gains have been achieved in reducing under-5 mortality, neonatal mortality has proven resistant to improvement and now accounts for 57% of all deaths of children under the age of 5 [2]. The vast majority of these deaths are preventable when women and families have access to and utilize routine and emergency maternal and newborn health (MNH) services [3].

However, in spite of considerable efforts by both the public and private health sectors, numerous barriers continue to prevent women and newborns from receiving these life-saving services, particularly in remote areas. The *three-delays model*, which identifies 1) the delay in deciding to seek appropriate obstetric care; 2) the delay in reaching appropriate services once the decision has been made; and 3) the delay in receiving services one at the health facility, remains highly relevant for analyzing factors contributing to low utilization of services in these regions [4]. The government of Bangladesh is committed to improving MNH and is currently implementing a strategy which aims to expand the provision of both routine and emergency services to even the most underserved regions [5]. Yet while the expansion and improvement of health services is essential to improving MNH and serves to address the third delay in particular, it is equally important to address the first two delays, and more globally to empower women, families and communities in order to assure that women seek and reach the care which they need. Overcoming these delays often requires tackling social, economic and cultural factors operating at the household- and community-levels.

Netrokona district, located in northern Bangladesh near the Indian border, is characterized by low socioeconomic status, a paucity of formal health services, and poor infrastructure. As a result, birth with the support of a skilled attendant, defined as an accredited health professional, such as a midwife, doctor, nurse or a community-based skilled birth attendant (CSBA)^a^, is even lower than the national rate. Only 10% of women give birth in the presence of a skilled attendant, compared to 24.4% nationally [6].

Against this backdrop, Participatory Action for Rural Innovation (PARI) Development Trust, a local non-governmental organization (NGO), supported by the Swiss NGO Enfants du Monde, has been implementing a program based on the World Health Organization’s framework for working with Individuals, Families and Communities (IFC) to improve MNH [7]. Grounded on the principles of Health Promotion, the overarching objectives of the IFC framework are to empower women, men, families and communities to improve MNH and increase access to quality health services. Interventions within the IFC framework aim to improve the care of women and newborns in the home and the community and to overcome the first two delays of the three delays model.

### The IFC program in Netrokona

Based on the IFC framework and in consultation with local authorities and community actors, the program in Netrokona district has focused on birth preparedness and complication readiness and working with traditional birth attendants (TBAs) to serve a new role in MNH which prioritizes education, referral and social support of women rather than birth attendance. These interventions aim to influence the social and cultural norms and practices surrounding care seeking in order to increase the utilization of skilled MNH services. Notably, prior to implementation of activities a situation analysis in 2005 revealed a general lack of planning for using skilled MNH services in anticipation of birth and potential complications. With few exceptions, women preferred birth with a TBA at home and did not recognize the value of the presence of a skilled attendant. Rarely were actions taken by women and families to prepare to access skilled care. TBAs considered themselves the principal provider of MNH services, as did women and families. Men generally felt that pregnancy and birth were a woman’s affair only and therefore remained hesitant to be involved, while still being the primary decision-maker, as the gate-keeper of family finances and women’s movement outside the household. All of these factors contributed to a low level of seeking and utilization of health services, and the program aims to influence these underlying factors to improve the health of women and newborns.

The program employs a rights-based approach as opposed to a needs-based approach to programming. Increasingly the international community has recognized that preventable maternal mortality and morbidity are primarily a result of the violation of women’s basic human rights; therefore, assuring that women have the opportunity to experience pregnancy and childbirth safely is not charity but rather a right to which they have legitimate claim [8-10]. The IFC program promotes women’s maternal health rights by increasing community awareness of women’s right to access quality maternal health services and by increasing women’s household decision-making authority so that they can autonomously seek care.

Implementation of the IFC program was initiated in 2008 by PARI Development Trust, a local nongovernmental organization (NGO), with the financial and technical support of Enfants du Monde, an NGO based in Geneva, Switzerland. Partners collaborating in the program include the Health and Family Welfare Department of Netrokona district and the following local NGOs: Jatio Tarun Sangha, Garo Baptist Convention/Primary Health Care Project and World Vision Bangladesh. Four of eight unions of Kalmakanda Upazila (sub-district) of Netrokona district were selected for implementation of program activities, namely Barkhapon, Rangchati, Nazirpur and Kharnoi, with program coverage in 80 of the 167 villages comprising these unions.

In order to promote birth preparedness and complication readiness, partner organisations worked with community representatives to elaborate a community awareness strategy including educational activities and support materials. Community- and facility-based health workers were trained to work one-on-one with pregnant women and their families to assist them in creating a plan and to work in groups with community representatives to build community awareness of the importance of birth preparedness and complication readiness.

A Birth and Emergency Preparedness Plan (BEPP) card was produced in collaboration with local partners. The card was developed by program partners according to WHO recommendations for birth preparedness and complication readiness, designed specifically for an illiterate or semi-literate target population. Prior to its utilization, the card was submitted to, approved and endorsed by the Health and Family Welfare Department of Netrokona district. This card illustrates the following preparations that women should consider in anticipation of birth: selecting a birth attendant; choosing a birth place and transportation to reach the birthplace; organizing with a birth companion; identifying a potential blood donor; developing a strategy to save money for costs related to pregnancy; and identifying where to seek care in the case of complications. This card also contains illustrations of the most common danger signs to help women and families identify complications and seek care in a timely manner. Women receive the card in one of two ways: from health care providers in facilities who distribute the card to women during antenatal care (ANC) visits and use it to help them develop a plan, or alternatively from CHWs who make home visits to women to provide them the card and help them to use it. This two-pronged strategy guarantees complete or near complete coverage of all women in the implementation site. The plan is then reviewed with the woman during all subsequent contacts with health workers during pregnancy.

After developing the plan with a health worker, women are encouraged to share it with their husbands and other influential family members, such as mothers and mothers-in-law. This exercise is intended not only to encourage women and families to choose skilled attendance at birth and seek skilled care in response to danger signs but also to empower women to participate in household decision-making processes, which is defined as their right [11].

As for the intervention pillar related to influencing the role of TBAs, meetings and workshops were conducted quarterly by program staff with TBAs, village doctors and homeopathic doctors to address defining new roles and responsibilities within MNH and discussing the importance of birth with a skilled attendant. They were also educated on danger signs in women and newborns and encouraged to refer and accompany women to health services in response to complications.

A midterm assessment of the program was conducted at the end of 2011, three years into the implementation of activities, in order to assess progress toward reaching program objectives. In this article we will review some of the results of this assessment to explore how the IFC program’s interventions are influencing social norms and practices related to MNH care seeking, specifically in the areas of birth preparedness and complication readiness and the role of TBAs and demonstrate how this, in turn, is promoting women’s rights related to maternal health.

## Methods

### Study design

The midterm assessment of the IFC program relied primarily on qualitative methods, with focus group discussions (FGDs) and semi-structured interviews forming the basis of data collection. Data was collected between October and December of 2011.

### Study sites

The study sites were selected from the IFC program’s implementation area comprised of the four unions of Barkhapon, Rangchati, Nazirpur, and Kharnoi, located in Kalmakanda upazilla. Two villages were selected from each union based on discussions with participating local NGOs and CHWs. As this study was conducted to evaluate changes resulting from IFC program implementation, selection of sites was based on the presence of program activities and distance from Kalmakanda. The rational was to ensure the reliability of data through proper representation of informants from all unions covered by the program. We considered the distance from Kalmakanda for ensuring researchers’ accessibility to participants that could otherwise be constrained by geographical barriers and seasonal adverse weather. Study sites are described in Table 1.

**Table 1:** **Study sites**

**Union**	**Village**
Barkhapon	Bishari
	Gaypur
Rangchati	Garam para
	Chikon tup
Nazirpur	Kuttakanda
	Rahimpur
Kharnoi	Gobindha pur
	Bamon goan

### Data collection

A team of five anthropologists, composed of one male and four female members with academic and professional backgrounds in social science research, conducted a series of FGDs and semi-structured interviews. The data collection team for the FGDs consisted of three members: one moderator and two members who simultaneously served as note-takers and gate-keepers. The semi-structured interviews were performed with individual participants by one interviewer. Female researchers moderated FGDs and semi-structured interviews with female participants and the male researchers conducted them with male participants. With the semi-structured guidelines used in the data collection, all the researchers had the freedom to ask follow-up questions about any new ideas or issues surfacing during the conversations for better understanding.

Two FGDs were conducted with pregnant women (defined as having completed at least 5 months of pregnancy) and women who had recently given birth (defined as within the previous 12-month period) and one each with following groups: husbands of women who were pregnant or had recently given birth, family members, TBAs, and health workers recruited from the partner NGOs as community health workers and facility-based health care providers. The focus group size varied from six to ten participants. In addition, semi-structured interviews were conducted with women who were pregnant or had recently given birth, family members of these women, health care providers, TBAs and CHWs.

All of the researchers were trained prior to approaching potential participants on predefined eligibility criteria for participation and on how to approach them to join as participants. Researchers obtained the contact information of women who had recently given birth from program registries and documents of partner NGOs after which they contacted them directly in the community. They set a time to conduct the FGD or interview with those who fit into the category and agreed to participate. No partner NGO staff were present during data collection procedures. Participants of the FGDs and semi-structured interviews are described in Table 2.

**Table 2:** **Methods and participants of the study**

**Group**	**Sample size**
**Method 1: focus group discussions**	
Pregnant women	1 FGD; 7 participants
Women who gave birth within past 12 months	1 FGD; 6 participants
Husbands of pregnant women/women who gave birth within past 12 months	1 FGD; 6 participants
Family members of pregnant women/women who gave birth within past 12 months	1 FGD; 6 participants
Traditional birth attendants	1 FGD; 9 participants
Community health workers	1 FGD; 6 participants
**Method 2: semi-structured interviews**	
Facility based health care providers	7
Traditional birth attendants	4
Community health workers	4
Pregnant women/women who gave birth within past 12 months	4
Family members/husbands	4 (2 family members; 2 husbands)

The instruments for data collection were developed in English and translated into Bangla, the local language. All interviews and discussions were recorded upon obtaining relevant consent from the participants. The duration of the FGDs ranged from 60 to 90 minutes and 45 to 60 minutes for semi-structured interviews. Homogeneity of the participants in terms of following characteristics was maintained: age, number of pregnancies, number of children, education, religion, ethnicity, local organization membership, and socioeconomic status. Demographic data were collected at the beginning of each FGD which are provided in Table 3. Key themes explored included knowledge and practices related to birth preparedness and complication readiness and awareness of rights related to MNH. Routine care seeking attitudes and behaviors were also examined (Table 3).

**Table 3:** **Demographic information of participants of FGDs**

**FGD participants**	**Sub-group**	**TBA**	**Pregnant mother**	**Delivered mother**	**Husband**	**MIL**	**CHW**
**Number of Participant**	-	09	07	06	06	06	06
**Education**	***Highest***	Class VIII	Class VII		Class X		
	***Average***	Literate	Literate	Primary level education	Class IX	No schooling	HSC
	***Lowest***	No schooling			Literate		
**Age (Years)**	***Highest***	53			30		
	***Average***	40–45	25–28	25–27	35–42	45	
	***Lowest***	40			45		
**Age at marriage**	***Highest***						
	***Average***		15–17	19–22	25		
	***Lowest***						
**Age at conception (Years)**	***Highest***						
	***Average***		25–27	19–20			
	***Lowest***						

### Data analysis

The data analysis followed a standard textual analysis process. All FGDs and semi-structured interviews were transcribed. Field researchers who participated in data collection were involved in transcription to ensure accuracy. Transcripts were then verified to ensure completeness. Bangla-speaking researchers coded a limited number of transcripts to prepare an initial list of codes following which inter-coder reliability was established. All the transcripts were then coded accordingly. We categorized data under themes and sub-themes from the list of codes describing similar kind of data and later compared and contrasted them. Data from the FGDs and interviews were then compared for data triangulation for ensuring reliability of data. A content analysis was conducted according to the method illustrated by Graneheim and Lundman [12]. The results were compared against the Situation Analysis of Maternal and Newborn Health: Netrokona District, Bangladesh 2005 conducted by PARI Development Trust and Enfants du Monde which was also carried out in Kalmakanda upazilla during the program planning phase.

#### Ethics

Ethical approval was obtained from the Ethical Review Committee of the International Centre for Diarrhoeal Disease Research, Bangladesh (ICDDR, B) prior to initiation of research. All ethical requirements were met to protect participants and their privacy. Written informed consent was obtained from each individual prior to their participation in the study during which they were informed on the scope, purpose, reporting, and method of maintaining anonymity in the study. The consent form and the research tools were translated into Bangla. None of the participants were given compensation or incentives for participation and this was stated in the consent form.

## Results

### Birth preparedness and complication readiness

According to study participants, planning for birth and emergencies is becoming common in the household, particularly in contrast to 2005 when this type of planning was virtually non-existent. Participants expressed that this change has been facilitated by utilization of the BEPP card which had been introduced in the study area by CHWs of partner NGOs. All participating women were aware of the BEPP card and had used one to elaborate a plan for birth and complications. Women had received their cards from CHWs participating in the program or from health care providers when having sought ANC in health facilities. The card was deemed to be effective in transmitting information to participants regardless of literacy status and illiterate men and women expressed an in-depth understanding of the elements illustrated in the card.

Notably, most of the informants from each category could mention all or some of the danger signs. However, in some cases, women and men mentioned complications that are not considered danger signs. According to women, danger signs include weakness, nausea, abdominal pain, acute problems with urination, feeling thirsty, vertigo, fever, presentation of hands and feet of baby during birth, convulsions, bleeding, high fever, more than 12 hours of labor, discharge with a foul odor and loss of appetite. Husbands stated danger signs for pregnant women as high fever, persistent severe headache, leaking membranes without labor pain, convulsion and bleeding. They also mentioned foul odor of urine, bleeding and severe headache as danger signs in women postpartum. As for newborns, they related low birth weight, jaundice for more than two weeks and fever as danger signs. In 2005, the knowledge of actual danger signs was almost absent among women and men Many of the husbands were illiterate and stated that they learned about these danger signs from the illustrations in the BEPP card. However, the fact that some participants stated signs that are not actually considered danger signs may suggest a need to refine the illustrations or clarify messaging.

While some husbands admitted that they had not looked inside the card, they stated that they had discussed the plan with their wives and were aware of and in agreement with the plan that had been prepared. One area in which it appears men had been actively involved was in planning for potential costs related to pregnancy and birth. Program staff had educated families on the costs that could be incurred from birth in a facility and from receiving emergency services. The majority of male participants had developed a strategy for saving money for these expenses which was in contrast to 2005, at which time families did not have any plan or savings for expenses related to birth or emergency services. One husband explained:

“*We collected everything beforehand and also saved money…[the CHW] advised me to save some money to get prepared if c-section was needed for [my wife’s] delivery…she also said that in 70% cases, women may need a c-section as my [wife] was weak…so it was better to make preparations beforehand…*”

Prior savings by the father of the child was found to be a primary source of funding for costs incurred for care-seeking in response to obstetrical and neonatal complications. This was in contrast to the previous practice of selling property and/or obtaining loans from someone with or without interest prior to the initiation of the program in the study area. In the case where the wife stayed with her parents during pregnancy, parents covered expenditures related to birth. Some families mentioned purchasing a small clay bank from the market to deposit change left over at the end of day, generally varying between 2–10 Bangladeshi taka. One husband from Rangchati explained:

“*I deposited 5 taka or 10 taka earned from working [daily]… this turns into 2000–3000 taka… this helped to manage the situation later.*”

While saving for birth and complications had become common, it was not clear how much money was generally saved and whether it was enough to cover the costs of the services.

All participants were aware of the importance of blood group screening, though several women had not done it or had waited until the later stages of pregnancy to do it. One reason stated for delaying or not completing blood group screening included fear of having blood drawn, though many women did not provide a specific reason. Regardless of knowing their blood group, all of the women had selected a potential blood donor prior to delivery. The situation analysis report from 2005 did not present any single case of blood screening during pregnancy or of having a blood donor ready for emergency in anticipation of birth.

All participating women had selected a birth attendant prior to birth. In the majority of cases a CSBA was preferred over a TBA to assist the birth at home, a stark contrast to the preference of women in 2005 at which time TBAs were nearly exclusively the birth attendant of preference. Both women and men expressed a strong preference for skilled birth attendants over TBAs, stating that skilled attendants possess unique skills and knowledge to preserve the health of the mother and infant that TBAs do not have. One newly delivered mother explained:

“*TBAs do not refer the mothers suffering from labor pain for more than 12 hours to the hospitals and say that it may not be needed… then the child dies in the womb… the mother dies… this kind of thing used to happen… and they know… the skilled birth attendants know… they send [women] to the hospital if the labor is more than 12 hours and if they can’t do anything…This is the reason for which we do not use the TBAs from the village.”*

While planning for birth and potential complications appears to be more common, barriers still exist for women and families seeking to implement the plan as in 2005. For instance, women are hesitant to seek services at formal health facilities since they fear that a male provider may ultimately attend the birth. According to the participants, this is culturally unacceptable. In addition, although all participants wished to give birth in the presence of a CSBA at home, availability of CSBAs was an issue. In situations where a CSBA was not available, the default action was to give birth with a TBA at home rather than going to a health facility, although most participants were aware that they had access to transportation schemes that had been developed within the context of the program.

### Role of TBAs

According the participants, TBAs remain highly influential in the community. Community members, health workers and TBAs themselves stated that they are trusted by women and families as they are local community members. While women and their family members generally expressed a growing preference for skilled attendants, TBAs still seemed to hold the authoritative position on matters relating to birthing care.

The workshops conducted within the context of the program for TBAs seem to have improved their understanding of danger signs or complications during childbirth and the need to refer women to facilities for receiving skilled care. For example, some TBAs mentioned that if the baby is in a breech position they immediately refer the woman to the health facility rather than attempting to manage the complication. One TBA stated:

“*I usually contact [the mother]. They call me for delivery. After I come, I call the doctor. I observe if they are healthy or sick. I try to find out if she is suffering from headache or fever, abdominal pain… after that I send her to the [family welfare visitor].*”

In general, TBAs expressed that they did not feel qualified to manage these types of situations and that they refer women to skilled providers when they present with these complications.

In addition to referring women experiencing complications, the majority of TBAs also reported encouraging women to seek skilled routine care. One TBA said:

“*I told [pregnant women] to do a monthly [antenatal care] checkup. I told them that if they cannot do a monthly checkup, then they should go at least once every two months. If they cannot do this, they should go 5 times in 9 months.”*

TBAs also reported accompanying women to the health care facilities for antenatal care, postnatal care and care at birth. TBAs said that some of the pregnant mothers showed reluctance in going to the facility as they were engaged with household chores and responsible for taking care of other children. However, they encouraged women to seek this care. According to one TBA:

“*I send them to the clinic. I take them with me. If [the woman] does not listen to me, and becomes arrogant and states that they have other activities (e.g. household activities) to do [rather than having a check-up], I [make them come] with me.*”

Most TBAs stated that they are well connected to CSBAs, health facilities and general practitioners. They expressed satisfaction with their experiences accompanying women to health facilities and feel that they are respected by health personnel. Some TBAs were provided a card to attest that they had participated in the workshops. They perceived that they were accorded increased legitimacy when presenting this card at health facilities.

Conversely, the majority of health care providers expressed general dissatisfaction with regard to TBAs’ involvement with women and reluctance to collaborate with them. According the majority of providers, TBAs give women harmful health advice, such as discouraging women from increasing their caloric intake during pregnancy in order to limit the growth of the baby under the assumption that giving birth to a larger baby would be difficult. Some providers in the public health facilities also mentioned that TBAs may be overconfident and therefore make harmful decisions. For instance, they stated that TBAs may bring women to health facilities only after having tried and failed to assist the birth, resulting in a more critical state for the woman. However, both many facility- and community-based workers agreed that these tendencies had been mitigated since the initiation of IFC program. According to them, TBAs should be more engaged in advocacy and dissemination of health-related information since they are close to the community.

TBAs overwhelmingly expressed enthusiasm in their participation in the program and a desire to work with partner NGOs to provide women information on attending antenatal care visits, postnatal care visits and household care of women and newborns. However, while the TBAs had participated in several orientation workshops, they could generally not recall the content of these sessions. They are eager to receive continued training and would like to receive instruction on appropriately identifying complications in women and newborns as well as on MNH generally.

### Maternal and newborn health rights

In general, women and men did not understand the concept of “rights” related to MNH. Body language and “blank” looks to each other in the FGDs exhibited their unawareness of terminology related to health rights. Women laughed and asked: “*Why should we have rights?*” Husbands were also unaware of rights related to MNH. One of the husbands said, “*Husbands give rights to the woman.*”

Though most of the women of this study were aware that they could receive certain maternal health services free of charge, they were generally hesitant to access these services, as they felt either they would not actually receive the services or that what they received would be of low quality. One woman described:

“*After I had been to the hospital, the doctors were not giving any medicine, didn’t come to visit me, I didn’t have that much money… I was almost dying. Later, we had to sell our cattle and brought the money there. If we didn’t give them the money, I would never have survived. After giving them the money, I got very generous care from them. The doctor came in every moment.*”

One woman went so far as to state that:

“*The more money one provides, the more care s/he gets.*”

Although participants made a link between paying for services and their quality, in general they expressed satisfaction with the privacy at health facilities, their interactions with health care providers and services received.

On a promising note related to rights, women and men expressed their perception that interventions related to birth preparedness and complication readiness are influencing the role of women in household decision-making processes which was previously limited to male members. Interactions between women and their family members in decision-making were reported to be present in terms of seeking health care from informal or formal providers. Though women’s participation in decision-making processes is reportedly increasing, informants also stated that women are still not the primary decision makers as the final decisions are made by the male members of the women’s family. One woman explained:

*“I told my husband [about trouble I faced] and asked him about where he would take me for care. I had to request him to take me with him… He arranged transport or sometimes the van to take me to the health care provider…My husband made decisions by himself… my mother-in-law also assisted him.”*

In contrast, in certain cases women stated that they would take action autonomously if their wellbeing and/or that of their infant required it regardless of what other family members said. On women stated:

“*What should one do!… if a person has to save her life, she may have to disregard others’ decisions, or they should be made aware of the situation… they should be told that there were no laws before and now there exists one… so we have to follow this now.”*

As communication with people outside the family has increased, such as when pregnant women organize with a potential blood donor or with drivers or owners of transportation, women’s capacity for care seeking and managing critical situations with more autonomy has also been augmented.

## Discussion

The findings of the present study suggest that interventions aiming to affect changes surrounding social norms and practices related to MNH in the household and community can set into motion the desired evolutions. One of the most promising findings is that it appears that interventions related to birth preparedness and complication readiness have resulted in an increase in preparation, which seems to be serving to reduce the delay in deciding to seek services. Evidence as to the benefit of birth preparedness and complication readiness as an effective intervention for increasing the utilization of skilled care and in improving MNH is now fairly well established [13-15], and, as such is included as an accepted intervention within antenatal care [16]. According to the situation analysis of 2005, prior to the implementation of the program women and families made few considerations in anticipation of birth and potential complications, with the exception of identifying a TBA to attend the birth at home. In contrast, this study revealed that there appears to be a more general trend now toward planning for birth and complications with participants crediting program interventions. Notably, the BEPP card had proven an effective tool for even semiliterate and illiterate populations and was well accepted by the community members, both men and women.

In addition, awareness of danger signs seems to be increasing among both men and women. However, both groups continue to cite some ‘signs’ which are not necessarily considered danger signs for which care should be immediately sought, such as nausea and feeling thirsty. This may be because they have confused some danger signs with common symptoms of pregnancy or postpartum. This suggests a need to reinforce communication on danger signs in the community, perhaps by also addressing common symptoms for which it is not necessary to seek care.

Moreover, there was a clear gap between awareness and action related to the identification of a blood donor in the case of postpartum hemorrhage. This is a critical action as hemorrhage remains the leading cause of maternal death and most local health facilities are not adequately equipped with a blood bank. While women were aware of the importance of planning ahead for this, some did not take these measures, and many waited until the later stages of pregnancy to prepare a blood donor. In addition to the fear of having blood drawn, this may be because there were not adequate resources available for completing the blood group-screening. Blood-group screening campaigns were conducted several times within the program, but this may not have been sufficient for the majority of women who sought no routine ANC during pregnancy and were therefore were not able to do this in a health facility.

Especially promising is the shift in birth attendant preference toward skilled care. This development is most striking in favor of choosing a CSBA to attend the birth at home, while barriers remain for women seeking facility-based care. It is estimated that providing skilled birth attendance at home could avert nearly half the number of stillbirths which occur globally and evidence has exhibited the potential of expanding skilled care to the community-level [17,18]. As such, Bangladesh Ministry of Health has made the strategic decision to include the expansion of CSBA coverage throughout the country as an axis of the national plan to improve MNH [5]. While it is unclear whether CSBA coverage will be a stopgap measure as birth is increasingly moved from the home into facilities or a long term solution, our findings suggest that meeting the need for CSBAs should be a priority as it has the greatest potential for increasing skilled attendance at birth in remote villages in the near-term.

In order to quantify the effect that birth planning has had on increasing skilled attendance at birth, a primary indicator of the program’s success, a quantitative study will be required. This study explored the issues and status regarding the process and perceptions of stakeholders on maternal health care. Participants expressed meeting continued barriers to skilled attendance, notably the lack of available CSBAs and continued social barriers to care seeking in facilities. To date there has been conflicting evidence regarding the impact of birth planning on realized skilled attendance at birth, with some studies finding an increase in skilled attendance attributable to birth preparation and other studies failing to find this correlation [13,15,14,19]. In Nepal, while birth preparedness was associated with an increase in knowledge, it did not impact the rate of skilled attendance at birth [13]. In contrast, studies in India and Uganda found that women who planned for birth were more likely to give birth with a skilled attendant [15,20]. However, it is not yet clear which elements of birth preparedness may be most critical for increasing skilled attendance. For example, a study in Burkina Faso determined that while certain components of a birth preparedness plan, such as saving money for birth, were associated with an increase in skilled attendance at birth, other factors, including planning to give birth with a skilled attendant, were not [21].

While the final evaluation of the program will measure whether the planning has resulted in a significant increase in skilled attendance, these qualitative results are already promising as planning across all components of a birth preparedness plan seem to be taking hold. Moreover, preference for skilled attendance was nearly absent in the situation analysis, with women and families almost exclusively choosing the services of TBAs. It is reasonable to hypothesize that these changes are translating into changes in the use of skilled care.

If TBAs are able to successfully serve a new role in MNH they may contribute to overcoming both the delay in deciding to seek services and the delay in reaching services. Limited evidence suggests that integrating TBAs into the health system can contribute to increasing skilled attendance at birth and improve MNH outcomes [22]. The fact remains that these unskilled attendants continue to assist the majority of births in the developing world and are very influential in communities. Our results suggest that TBAs are generally receptive to assuming a transition in their role, and are willing to persuade women to use skilled care for antenatal and postnatal care visits as well as during birth, and to refer women with complications to an adequately equipped health care facility. These attendants expressed a great desire to make the best decisions to improve their clients’ health. However, this pillar of the programme will need to be strengthened as TBAs were overwhelmingly unable to recall to content of the workshops they had attended. In addition, it will be critical to identify a way for TBAs to be remunerated for the work that they do outside of assisting birth to compensate for the loss of wages associated with birth attendance.

In addition, in order to optimize the potential of TBAs, greater effort is required in targeting the health services, as they will be more likely to be successful in working in a new role if they have the support of health care providers and are betting integrated within the system. The staff at health facilities may serve an important function in encouraging this new and important role of TBAs, which is likely to be an uphill task since not all providers currently hold a favourable attitude toward collaborating with TBAs. In response to these observations, workshops will be held with both health care providers and TBAs to discuss their new role and meetings will be held between the two groups to facilitate integration of TBAs into the health system.

Finally, while placing maternal health within a rights context has gained momentum in the international community throughout recent years, this study highlighted some of the challenges of translating this theoretical framework into change at the ground-level. The concept of rights in Netrokona district is poorly understood generally, much less women’s rights or rights related to maternal health. While women know that they should be able to access certain maternal health services free of charge, they are hesitant to do so as they feel that the quality will be low.

However, it is promising to note that interventions related to birth preparedness and complication readiness seem to be contributing to promoting women’s rights related to maternal health by increasing their influence over decision-making processes. Although women are still generally not the final decision makers, these data reflect a change from earlier analyses during which it was expressed that decisions related to MNH were almost exclusively made by men and, in some cases, mothers-in-law, with an absence of participation by women. As women worked privately with health workers to develop a plan for birth and emergencies, which they later shared with other household members, they became the household authority in birth preparedness and complication readiness.

Women appear to be discussing their plan for birth and potential complications with their husbands and family members, which has initiated a practice of involving pregnant women in decision-making and valuing them for the knowledge they have acquired. Though women’s participation in decision-making processes was present in most cases, they are still not the primary decision makers as the final decisions are made by the male member(s) of the women’s family. However, in emergencies, women are being empowered to make decisions for the wellbeing of themselves and that of their infant. It was also found that ensuring transportation beforehand to reach the health facility, choosing a person to accompany the woman and savings for emergencies also reduced dependency on male members of the family and is now allowing women to act more autonomously.

It is important to note that the results of this study suggest a greater change related to the first delay in deciding to seek care compared to the change in accessing care. This may indicate a lapse time between building the awareness required to overcome the first delay and the subsequent step in utilizing means available to seek services. It is reasonable to expect that a certain amount of time would be required to anchor the necessary knowledge and decision-making processes before women and families take the next step in using the means available to access services. Alternatively this may suggest that the current solutions put in place to overcome the second delay are not sufficient. Future evaluations will explore this in more depth.

### Limitations

An important limitation of this study is the risk of potential response bias. There is a risk that participants have provided the perceived desirable responses, for example regarding support for the project or TBAs reporting on the degree to which they advise women to utilize health services. It will be important when carrying out future evaluations to further triangulate data to account for this risk.

An important limitation of the IFC program is that it ensures awareness building and capacity development to seek care in emergency; however, it does not provide direct health services to the people living in the remote areas. This remoteness may further contribute to discourage pregnant women to seek routine care from the facility providers or seek health care due to distance and inconvenient communication system. Future phases of the program will strengthen health services at the community-level through provision of CSBAs and through advocacy for the deployment of skilled health professionals at the health facilities.

## Conclusion

The findings of this present study suggest that community-based programs aiming to influence knowledge and practices can successfully initiate changes in social norms and practices related to MNH. As a result of the IFC programme in Netrokona district, women and families are beginning to plan for birth and potential complications and TBAs have started to serve a new function in MNH. Women are also participating to a much greater degree in decision-making processes. These developments are expected to contribute significantly to addressing the first two delays of the three delays model: the delay in deciding to seek care and the delay in reaching care. Future evaluations will serve to quantify the impact of program interventions and how it has contributed to the utilization of MNH services to improve the health of women and newborns.

## Endnote

^a^CSBAs are birth attendants trained over a period of 6-months in the essentials of care during pregnancy, birth and during the postpartum period and then placed directly in communities to provide services in women’s homes, or in community clinics. These health workers are able to manage normal birth and are trained to refer women and newborns with complications to health facilities. They are included as part of the current national MNH strategy in Bangladesh.
